# Editorial: Mechanisms of action of small molecules on CFTR mutants and the impact on cystic fibrosis pathogenesis

**DOI:** 10.3389/fmolb.2024.1446875

**Published:** 2024-06-24

**Authors:** Camille Ehre, Miquéias Lopes-Pacheco, Onofrio Laselva

**Affiliations:** ^1^ Marsico Lung Institute, University of North Carolina at Chapel Hill, Chapel Hill, NC, United States; ^2^ Department of Pediatrics, University of North Carolina at Chapel Hill, Chapel Hill, NC, United States; ^3^ Department of Microbiology and Immunology, University of North Carolina at Chapel Hill, Chapel Hill, NC, United States; ^4^ Department of Pediatrics, Emory University School of Medicine, Atlanta, GA, United States; ^5^ Center for Cystic Fibrosis and Airway Disease Research, Emory University and Children’s Healthcare of Atlanta, Atlanta, GA, United States; ^6^ Department of Clinical and Experimental Medicine, University of Foggia, Foggia, Italy

**Keywords:** cystic fibrosis, CFTR modulator therapy, personalized medicine, genetic disease, mutation specific

Cystic fibrosis (CF) is a complex, life-threatening genetic disease that has witnessed a profound transformation in the treatment landscape over the past decade (Oliver et al., 2023). Historically, the disease has been managed through symptomatic treatments, but the development of highly effective CFTR modulators therapies has launched a new era targeting the fundamental cause of the disease – the defective CFTR protein. However, despite significant therapeutic advancements, ongoing challenges highlight the need for continued research and personalized approaches.

The aims of this Research Topic “Mechanisms of Action of Small Molecules on CFTR Mutants and the Impact on Cystic Fibrosis Pathogenesis” are (1) to target the underlying molecular defect in CF, (2) to enhance the functionality of the CFTR protein, thereby improving respiratory and digestive functions, as well as the overall health and quality of life for people with CF (pwCF), (3) to expand treatment options for rare CFTR variants, (4) to investigate the long-term impacts of CFTR modulators, particularly regarding drug safety profiles and potential adverse effects on liver function and other aspects of health, and (5) to explore RNA-targeting strategies, such as antisense oligonucleotides (ASOs) and small molecules that target RNA interfaces, aiming to expand the therapeutic landscape for CF and potentially other related genetic disorders.

The introduction of CFTR modulators marked a groundbreaking shift in CF therapeutics. These small molecule compounds, categorized as potentiators and/or correctors, are designed to rectify the dysfunctional CFTR protein. Potentiators like ivacaftor (VX-770 or Kalydeco^®^) enhance the function of CFTR proteins at the cell surface (Van Goor et al., 2009). Correctors such as lumacaftor (VX-809) and tezacaftor (VX-661) facilitate the proper folding and trafficking of these proteins (Van Goor et al., 2011). Elexacaftor (VX-445) is an alternative corrector that also displays direct potentiator effects (Laselva et al., 2021). The combination therapy of elexacaftor/tezacaftor/ivacaftor (ETI, also called Trikafta/Kaftrio^®^) has shown remarkable success, particularly for pwCF carrying at least one F508del allele, the most prevalent CF-causing variant. This triple combination therapy has significantly improved lung function and quality of life for many patients with CF (Bacalhau et al., 2023).

One of the goals of this Research Topic is to highlight the real-world impacts and clinical outcomes of highly effective CFTR modulators. To this end, the meta-analysis described by Xu et al. underscores the efficacy of ETI therapy, reporting substantial improvements in pulmonary function and nutritional status, alongside a decrease in sweat chloride levels – a key biomarker of CFTR function. The safety profile of ETI is generally favorable, with severe adverse events occurring rarely. However, despite these positive outcomes, the incidence of mild and moderate adverse events remains high, necessitating ongoing monitoring and management.

A fundamental principle of CF research is that pwCF respond differently to CFTR modulators, even for those carrying CF-causing variants with similar primary defect(s). The case study by Allan et al. underlines a critical challenge for CF clinicians and patients: the variability in response among patients with rare CFTR variants. For instance, a CF individual with the Q1291H/F508del genotype showed no benefit from ETI therapy and experienced adverse effects, illustrating the need for personalized treatment strategies. In silico and biochemical analyses revealed that such rare variants can have unique impacts on CFTR structure and function, emphasizing the need for patient-specific drug response testing.

Balancing efficacy and safety remains a significant issue in the development of CFTR modulators to ensure that treatments not only improve patient outcomes but also minimize adverse effects. The study by Castaldo et al. instills a nuanced view of the long-term effects of CFTR modulator therapy. While lumacaftor/ivacaftor (LI) and subsequent ETI treatment significantly improve BMI and lung function, ETI is associated with increased liver enzyme levels, indicating potential liver function impairment. This finding calls for cautious monitoring and further research into the long-term hepatic effects of ETI.

Expanding the therapeutic horizon, RNA-targeting strategies offer exhilarating new avenues for treating cystic fibrosis and other genetic disorders by addressing disease mechanisms at the molecular level. Complementing the progress in small molecule therapies, the review article by Li et al. discusses the rapidly expanding field of RNA-targeting treatments. These approaches, including antisense oligonucleotides (ASOs) and small molecules targeting RNA interfaces, offer hope for a range of diseases, including CF. However, challenges such as high costs, delivery issues, and selectivity need to be addressed to realize the full potential of these therapeutic approaches.

The future of CF research holds great promise through personalized and collaborative approaches aimed at enhancing treatment. To navigate the complexities of CF treatment, a multifaceted approach is essential. Personalized medicine, using patient-derived tissues for testing drug efficacy, can tailor treatments to individual needs, addressing the variability in mutation-specific responses. Advanced screening techniques, including high-throughput and *in silico* screening, can accelerate the discovery and optimization of novel modulator compounds. Comprehensive clinical trials seeking to expand and include diverse CF mutations will need to provide robust data on the efficacy of therapies across the CF spectrum. Furthermore, collaborative research, involving partnerships between academia, industry, and patient advocacy groups, can drive innovation and ensure alignment with patient needs. This holistic approach will be instrumental in enhancing the development and application of effective CF treatments.

In conclusion, the combination of CF modulators has revolutionized CF treatment, offering unprecedented improvements in disease management and patient outcomes. However, the journey is far from over. Continued research, personalized treatment approaches, and collaborative efforts are crucial to extending the benefits of these therapies to all pwCF, regardless of their genetic mutations. As we move forward, the promise of small molecule therapies in CF remains an encouraging prospect, driving us towards a future where CF can be managed more effectively, and perhaps eventually, cured.
